# Social values and sustainable development: community experiences

**DOI:** 10.1186/s12302-022-00641-z

**Published:** 2022-08-09

**Authors:** Walter Leal Filho, Vanessa Levesque, Subarna Sivapalan, Amanda Lange Salvia, Barbara Fritzen, Ronald Deckert, Valerija Kozlova, Todd Jared LeVasseur, Kay Emblen-Perry, Ulisses M. Azeiteiro, Arminda Paço, Bruno Borsari, Chris Shiel

**Affiliations:** 1 Department of Natural Sciences, Manchester Metropolitan University, Chester Street, Manchester, M11 5GD UK; 2 Department of Environmental Science and Policy, University of Southern Maine, 106 Bailey Hall, 37 College Ave, Gorham, ME 04038 USA; 3 School of Education, University of Nottingham Malaysia, Selangor, Malaysia; 4 Graduate Program in Civil and Environmental Engineering, University of Passo Fundo, Campus I - BR 285, Passo Fundo, São José, RS 99052-900 Brazil; 5 Dekan Fachbereich Technik, HFH · Hamburger Fern-Hochschule, Alter Teichweg 19, 22081 Hamburg, Germany; 6 Faculty of Business and Economics, RISEBA University of Applied Sciences, Meza Street 3, Riga, 1048 Latvia; 7 College of Charleston, School of Humanities and Social Sciences, Sustainability Literacy Institute, Charleston, USA; 8 Department of Management and Finance, Worcester Business School, University of Worcester, Worcester, UK; 9 Department of Biology and CESAM – Centre for Environmental and Marine Studies, University of Aveiro, 3810-193 Aveiro, Portugal; 10 NECE-UBI (Research Centre for Business Sciences), Universidade da Beira Interior, Rua Marquês D’Ávila e Bolama, 6201-001 Covilhã, Portugal; 11 Department of Biology, Winona State University, Winona, MN 55987 USA; 12 Department of Life and Environmental Science, Bournemouth University, Fern Barrow, Poole, BH12 5BB UK; 13 European School of Sustainability Science and Research, Hamburg University of Applied Sciences, Ulmenliet 20, D-21033 Hamburg, Germany

**Keywords:** Social values, Sustainability, Community, Projects, Initiatives

## Abstract

**Background:**

This paper presents a review of the literature and trends related to social values and sustainable development and describes a set of case studies from a variety of community-based projects which illustrate the advantages that social values bring about as part of efforts to promote sustainability. Three approaches were used to develop this study: a bibliometric analysis of the topic “social values and sustainable development”, an analysis of case studies that concretely present community projects addressing social values and sustainability, and the development of a framework linking up bibliometric clusters and the cases studies.

**Results:**

While the bibliometric analysis revealed clusters where social values are strongly connected with sustainable development, the case studies indicated the lack of a common terminology and understanding of the relation between social values, sustainable development, and community-based projects.

**Conclusions:**

The study concludes by suggesting a set of measures that could be deployed to better take social values into account when planning policies or making decisions related to community projects.

## Introduction

September 2015 marked a significant milestone for the people, planet, and prosperity. It was during this historic occasion that United Nations Member States collectively agreed upon the adoption of the Sustainable Development Goals (SDGs). The adoption of the SDGs would see the global population come together to realize the urgent call for action to end poverty, safeguard the planet, and ensure peace and prosperity. The SDGs consist of 17 interlinked global goals that are designed to serve as a blueprint to achieve a more sustainable future for the global community, addressing among others, critical issues such as poverty, quality education, climate change, clean water and sanitation, partnerships, and sustainable communities. The emphasis of the SDGs and Agenda 2030 on addressing the dimensions of people, planet, prosperity, peace and partnership is the further assertion that these aspects are crucial to the future of humanity and the planet [56].

Agenda 2030 and the SDGs are essentially a socially driven agenda, projecting—social values and trajectories. To be able to better understand the place of values within the sustainability debate, there is first a need to define social values in this context. From a sociological perspective, values are considered the foundation for the spurring of human actions. Values are also deemed to be instrumental in the development of an individual’s personal and collective identities, besides being a vital conduit for social integration [54], while being appreciated by those focused on sustainable business. According to the organization Impact [29], a social value is seen as a strategic and achievable process that involves impacting societies positively, regardless of an entity’s financial status, business direction, or size.

In a sustainable development context, values are often considered in the assessment of communities classified as vulnerable [51]. Under-development, environmental ethics, and preservation of social and cultural traditions are but some of the prevalent issues explored in the literature on this subject matter [57]. Development has had an impact on the economy and the environment. This situation generates a critical purpose to investigate, while defining and evaluating the value of development, particularly from a social stance [25].

Social values never occur in a vacuum. We are socialized into pre-existing yet malleable conceptions of community and social relations [28, 40]. At the same time, there is a tension between autonomy and egotism, the need to create a healthy sense of individual self can conflict with the need to maintain a healthy and coherent community, with established social mores. Historically, human communities have created and policed social values that have privileged the latter—for example the hierarchical “5 great relationships” of Chinese dynasties informed by Confucianism, or the varna (caste) system of India. With the onset of violent European clearances, global colonialism and industrialization, coupled with individual is facilitated by the Protestant Reformation, the strong bonds of community values (that were also patriarchal, heteronormative, and confining for many), were sundered. This inversion of the social order flourished in the post-World War II US economy. It was based on limited affluence where individual consumers became the social model of modernism, establishing a culture that diminished all other relations. This social value of extreme individualism has in large part become globalized and has led the 2000s to anomie, dysnomia, economic crime [39, 44], and planetary crises. It has also led to high rates of mental health, suicide, and life dissatisfaction for many, where these factors are compounded by living through environmental devastation brought by the same system of over-consumption and over-population [46]. It is in this context that the focus on social values related to healing, flourishing, and justice as well as mutual support and a sense of community become central to sustainable development and a social leg of sustainability; which is related to personal growth and being connected [28]. Therefore, all people could aim at „personal evolvement in the community “ the as English translation of the German expression “Persönliche Entfaltung in Gemeinschaft” [14], 32).

We point out, though, that social values in support of sustainable development rightfully focus on equity, inclusion, and justice, but more and more data suggest that such values must also focus on and include the natural world and the connection of humans to it, as well [53]. Additional data indicate that as long as rampant individualism and a strong anthropocentrism tethered to values of over-consumption continue to shape social values and patterns of behaviours [33], sustainable development will be very hard, if not impossible, to achieve. Thus, the question arises about what social values are being advanced that can promote sustainable development? For example, values that (1) strengthen resilience; (2) support change and transformation; and (3) advance a social basis for these two conditions that may support sustainable development. Values that activate thinking, feelings and actions and that relate to determining benign change [49] may act to overcome dysfunctional norms and values humans learned to follow in societies and communities.

Interestingly, there has not been much literature focusing on the notions of social values and sustainable development within the context of community experiences [22, 48]. Thus, with this paper we aim at creating an avenue to explore these concepts in greater depth. More specifically, we aim to understand the extent to which the notion of social values and sustainable development have been approached and described within literature, to draw out international best practice case study examples showcasing social values and sustainable development within community-based projects, and to develop a framework integrating the best practice case studies and literature analysis.

The theoretical underpinning of our work is a three-pronged framework that considered stakeholder theory to understand how economic value is created and traded, including its links to ethics and capitalism. According to Parmar and his collaborators [45] this knowledge is necessary to assist entrepreneurs to reflect about management with emphasis on the value of goods and trading practices. Institutional theory instead is a paradigm about the more profound aspects of social structure, that focuses on the processes by which schemes, rules, and norms, become established to guide social behaviour [2]. The third prong of our framework is the point of convergence of the previous two, consisting of the theory for sustainable development as proposed by Shi and team [50]. This process is an evolutionary path that began with the single goal of using sustainably Earth’s resources, to Millennium Development Goals (MDGs), and most recently, the Sustainable Development Goals (SDGs). We first present a review of literature and trends related to social values and sustainable development. Following there is a discussion on a set of case studies from a variety of community-based projects which illustrates the advantages a focus on social values can bring about in promoting sustainable development. Finally, conclusions are made and some measures are listed, which may assist in deploying a better understanding of social values into account, when planning policies, or making decisions on spending, for which the sustainability of specific groups and communities may be jeopardized.

## Methodology

We are interested in exploring the context in which community-based projects focused on sustainable development have explicitly assessed and incorporated social values. One method of doing so is to analyse the publications about these topics, to assess the linkages and themes within this research area. Our methodological approach occurred in three main steps:

- Step 1: Bibliometric analysis of the topic “social values and sustainable development”.
- Step 2: Cases studies that concretely present community projects addressing social values and sustainability.
- Step 3: Framework connecting bibliometric clusters and the case studies.

Firstly, we conducted a bibliometric analysis using the software tool VOSviewer. This analysis allowed us to assess scientific investigation by using quantitative studies; it is based on the assumption that the number of citations of an article tends to reflect its impact on the scientific community [59]. Bibliometric analyses generated information about the quantity and performance of the publications, giving insights into the relations between fields of knowledge by means of the statistical analysis of co-publications and citations [47].

This bibliometric analysis included peer-reviewed publications indexed in the Web of Science (WoS). This is one of the most trusted and well-known worldwide citation databases covering multidisciplinary research. The following search string was used: TOPIC: (“social values”) AND TOPIC: (“sustainable development”). All years of the timespan available at WoS were considered (1945–2021). Only studies in English were considered, with no restrictions applied regarding document types. The search was carried out on March 2021 and returned 89 papers. In a second step, the titles and abstracts of the identified papers were checked, in order to validate their relevance and ensure their compatibility with the aims of the study. The exclusion criteria used in the study were: thematic relevance, interdisciplinarity, due emphasis to social science components under the lenses of sustainability. Based on these criteria, 74 articles were chosen for analysis. The co-occurrence analysis was performed in VOSviewer and returned a set of nodes and links. Each node is a frequently used term in the articles (analysed in titles and abstracts) and the size of the node refers to the frequency of the keyword. The distance between two nodes indicates the strength of the relation between the terms; therefore, shorter distances tend to suggest stronger relations [37]. Linked topics mean they have appeared together, and the link width is proportional to the number of co-occurrences the keywords have [37, 47]. The minimum number of occurrences of a keyword was set to 2, resulting in 24 selected keywords. For the process of clustering, where the software grouped closely related nodes in clusters, 2 terms were defined as the minimum number of keywords per cluster and clustering resolution was set to 0.5 (as per the software guidelines, this parameter determines the level of detail of the clustering and must have a non-negative value; the higher the value, the larger the number of clusters produced).

The bibliometric analysis was complemented by a qualitative assessment of the literature that focused on a set of community projects that incorporated social values for sustainable development. In this second phase, from the results of the bibliometric analysis, we presented selected case studies of community-based projects and initiatives. Keywords addressing social values such as: equity, inclusion, justice, human rights, health, values and life quality, were considered to identify the cases, as suggested by Estes [17]. More constructs were taken into account, such as: strengthening, resilience [10], and support for change and transformation [32]. Worldwide initiatives were analysed considering the following questions: what were the project’s goals? To which results has it led, in a sustainable development context? What difference is the project making? The collected case studies were presented in a summary table containing information about the title of the initiative, the goal of the project/programme, the main results, its geographical location and a reference article.

Each reported case study was examined and assessed for its consideration of sustainability topics such as communities’ resilience, social inclusion, gender equality, eco-innovation, and for how those topics intersected with each of the four clusters that emerged from the bibliometric analysis.

For Step 3, the analysis of the results from the previous 2 steps, or phases, served as the foundation for the development of a framework, which associates the case studies (Step 2) with the clusters identified in the bibliometric analysis (Step 1).

## Results

### Bibliometric analysis

The bibliometric analysis of the 74 selected articles showed that the publication on the topic of social values and sustainable development is still incipient (first publication dated 1992) and with over 60% of the publications occurring in the last 5 years.

**Fig. 1:**
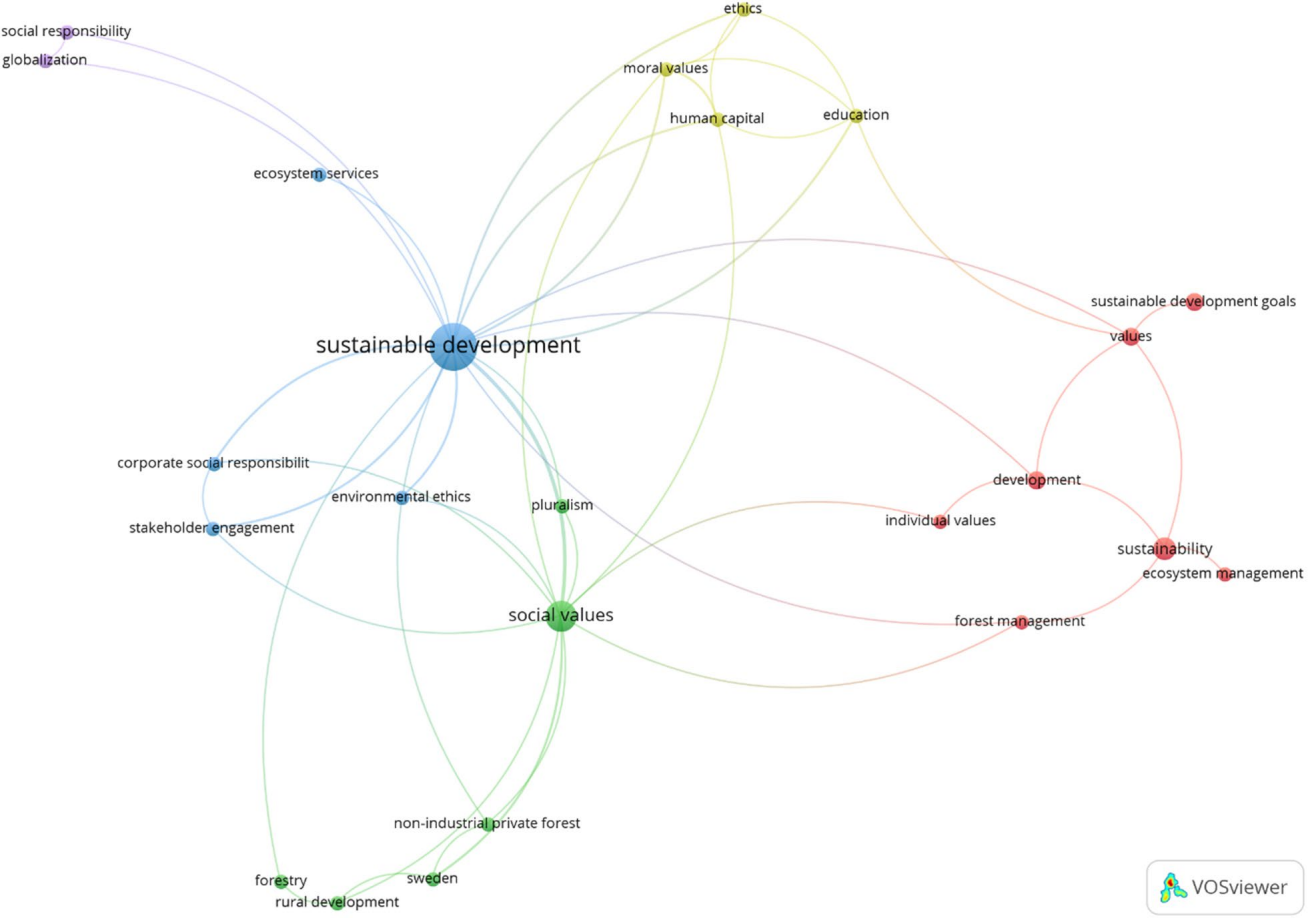
Output of the co-occurrence analysis

Results of the term co-occurrence analysis are presented in Fig. 1

and illustrate the main topics associated with social values and sustainable development derived from the literature. Even with a modest set of references resulting from the applied search string, four primary clusters were generated from this initial classification.

The red cluster centres on ecosystem management, such as forest management, incorporating explicitly, social values and sustainable development. Ecosystem management is a concept feature that aims to protect environmental conditions by taking into account the larger ecosystem context, as well as sustainable development and thus, is inclusive of social values and needs [35]. Similarly, sustainable forest management is a practice in which protecting and maintaining forests’ values is balanced with forests’ sustainable development in a way in which various, sometimes competing, social values come into play [6, 26].

The green cluster is focused on rural development, such as privately owned forestry, which suggests that some sustainability development researchers could be interested in the social values of rural development settings. For example, studies from Sweden and China revealed that while rural development efforts can promote sustainable development, there is often an underlying tension between urban and rural social values, suggesting a need to identify more explicitly, the role of and impacts on social value systems in rural areas [6, 27].

The blue cluster centres on business sustainability, integrating concepts such as corporate social responsibility, stakeholder engagement (in a corporate setting), and ecosystem services. Businesses have a role to play in achieving sustainable development, and research has explored the ways in which corporate social responsibility is driven by company and stakeholder social values, as well as the challenges of creating an economically viable business while maintaining core values [16, 20, 60]. The small purple cluster is an offshoot of the business-centric blue cluster, with a more specific focus on social responsibility and globalization. For example, a study from Croatia explored the intersection of and tension between the dynamic changes in local economies due to globalization, especially regarding the social values and identity of rural communities that create indigenous products [15].

Finally, the yellow cluster centres around ethics and moral values. There is much interest in the degree to which ethical and moral values influence environmental attitudes, human capital, adoption of sustainable practices, and transitions to more sustainable futures [11, 12, 24, 41]. These social values might arise out of religious [12], educational [3], or neighbourhood settings [41].

### Case studies

Multiple case studies on social values for sustainable development in community-based projects could be found around the world. Table 1

**Table 1:** Case studies on social values for sustainable development in community-based projects

Title of the initiative	What were the project’s goals?	To which results has it led, in a sustainable development context? Which difference has it made?	Location	References
Building Resilient Communities to Support the Health and Wellbeing of Venezuelan Refugees	This innovative project proposes a set of technological tools and resources to integrate urban planning and management of resilient communities for Venezuelan refugees in terms of accessing health and well-being support in Brazil and Colombia	It contributes to the development of effective public resilience policies and practices in response to COVID-19. These tools seek inclusive interventions, of a technological nature, that offer support to public agencies to assist and support the health and well-being of this vulnerable group	Brazil and Colombia	LabCom—UFPel [34]
Desa Makmur Peduli Api (Prosperous and Fire Free Village)	This initiative is focused on two activities: The Fire Management System Program and the Community Empowerment Program. It aims to reduce forest fires, create resilience, enhance food security, and alleviate poverty	Frequent forest fires cause damage and losses in terms of health, environment, society and economics and have a negative impact on extractive activities (timber, palm oil, and other commodities). This programme is successful to reduce the fire hot spots and provide additional income monthly	Islands Sumatera and Kalimantan in Indonesia	Pasaribu et al. [43]
Equator Initiative	The programme recognizes and advances local sustainable development solutions for people, nature and resilient communities through three action areas: Equator Prize, Equator Dialogues, and Equator Knowledge	It provides opportunities for indigenous people to address the challenges of land degradation, biodiversity conservation and livelihood improvement in a socially equitable manner	Equator	Berkes and Adhikari [5]
Enabling Communities for Climate Change Adaptation Planning	The project mainly links SDG 5 and 13 to empower local communities through Community based Organizations and youth ambassadors to design local climate change adaptation plans. It will also provide a platform for local communities to share these climate change adaptation plans with relevant ministries and municipal councils	It helped in augmenting the capacity of civil society and youth in three climate change hotbeds by a set of interventions which addressed a better understanding of climate change science and the ability to craft and deliver gender-sensitive adaptation plans. It empowered civil society and youth with the knowledge and skills to develop effective gender-sensitive strategies for climate change adaptation in their areas	Jordan	United Nations [55]
Enhanced Rural Resilience in Yemen (ERRY)	This initiative’s aim was to support displaced marginalized, youths and women to establish decentralized solar energy systems to improve access, employability skills, stable income and self-confidence	The distinguishing feature of this initiative is to make solar energy accessible and affordable to all. Solar micro businesses have recovered 50% of the seed grant in addition to a $100/month stable income since the establishment of the business	Yemen	Abyan et al. [1]
Farmer Managed Natural Regeneration (FMNR)	This is an easy and low-cost land restoration initiative to combat poverty and hunger amongst poor subsistence farmers by increasing food and timber production and resilience to climate extremes	It is both an effective climate mitigation and adaptation intervention where farmers can protect and manage the growth of trees and shrubs that regenerate naturally. It addresses multiple problems simultaneously through the restoration of vegetation, such as: land degradation, food insecurity, drying of springs, etc.	African, Asian and the Caribbean countries	Kandel et al. [31]
Food Assistance for Assets (FFA) for Resilient Communities in Latin America and the Caribbean	This initiative is aimed at improving, and providing cash, voucher or food transfers	In 2018, more than 230,000 people directly benefited from its programmes with 5,500 hectares of land rehabilitated, 290 water ponds, shallow wells, and fish ponds built, 155 km of feeder roads constructed or repaired, 5,800 social or community infrastructure assets constructed or rebuilt	Guatemala, El Salvador, Colombia, Honduras, Haiti, and Bolivia	FAO and WFP [19]
GO-GRASS	Based on harnessing regional assets, this project aims at diversifying, revitalizing and strengthening rural economies with quality jobs and opportunities in cooperation with entrepreneurs and local authorities	It has developed a set of cost-effective and sustainable circular small-scale bio-based solutions and business models to unlock the overlooked potential of grassland across European communities and create new business opportunities for rural areas	Sweden, Germany, the Netherlands, and Denmark	Go-Grass [23]
INCLUDE (Indigenous Communities, Land Use and tropical Deforestation)	This project details the economic, social and cultural damage being inflicted by tropical deforestation and pushes for change, empowering indigenous communities for decision-making and economic gains through sustainable agricultural modes of production	It addresses issues of governance, power and injustice, and involves the perspective of marginalized groups like indigenous people	Chaco Salteño—Argentina	European Union [18]
IOF2020 – Internet of Food and Farm 2020	This initiative brought several projects addressing IoT (Internet of Things) in the sustainable agri-food sector (meat, arable, dairy, vegetables and fruits) in different European communities	It fostered a symbiotic ecosystem of technology providers and players, helping to accelerate the virtuous cycle of adoption and maturation of IoT in the agri-food section, making European communities and farming more competitive	Europe	Sundmaeker et al. [52]
Microsoft’s 4Afrika Initiative	Since 2013, this initiative aims to unlock and accelerate Africa’s potential to create technology not only for the continent, but for the world	It creates investments in startups, partners, small-to-medium enterprises, governments and youth. Significant strides across key economic sectors, including agriculture, social impact, healthcare and skills development have been made in Africa	Africa	IFC et al. [30]
Natura & Co Commitment to Life	This is a 10-year timeframe programme to address the climate crisis and protect the Amazon, ensuring equality and inclusion, and shifting our business towards circularity and regeneration	Some targets were established to protect the Amazon forest (zero deforestation, to expand influence to 40 communities, to share at least R$ 60mi in value with communities), to defend human rights (to gender balance and equitable pay), and to embrace circularity and regeneration (95% + renewable or natural ingredients and biodegradable formulas)	Brazil	Nature and Co [42]
NEIGHBOURHOOD CHANGE	This project looked to community-based initiatives for inclusive solutions. It aimed to connect local government flexibly and maximize community engagement, promoting change	It has repositioned local authorities at the centre of social innovation debate, and called for more flexible and transparent planning systems, open to collaboration between community-led initiatives and public administrations. Recently, its findings are being further explored as part of the SOLIVID project to develop a collaborative map and online resource of the solidarity initiatives set up in response to COVID-19	Italy	Clark and Coulter [13]
Protege BR (Protect Brazil)	This initiative connects the needs of the public health centres with the manufacturers of local products and technologies, solving health problems in the rural and small communities	Recently, it worked to increase the number of health professionals using personal protective equipment during the COVID-19 pandemic, in areas far from large centres in Brazil, to reduce a large number of workers on sick leave due to contagion	Brazil	Braida and Unanue [9]
Re:Code – Lego Group	Re:Code is part of the Lego Group Local Community Engagement Program. Using Lego Education products, it consists of fun learning events gathering children to create, invent and code robotic models that solve real issues, mostly around sustainability	These events host hundreds of children to participate in activities that deepen learning on real-world themes while helping to boost twenty-first century skills	26 countries	Lego et al. [36]
Red Rocks Initiative for Sustainable Development	This initiative promotes projects to enhance sustainability in the Virunga Mountains, such as the Agritourism, Livestock Support for small-scale dairy farmers, Agriculture Project for small-scale subsistence farmers, Igihoho Project (biodegradable bags made of banana bark), Women’s Weaving supporting the widowed single mother in banana bark weaving activities, among others	It aims to ensure the sustainable social and economic development of communities by supporting locally led environmental conservation and sustainable tourism initiatives	East Africa countries	Bakunzi et al. [4]
Social Fuel Seal—BSBios	Since 2007, BSBios, a Brazilian biodiesel company, holds the Social Fuel Seal, acquiring biodiesel raw material (soybean, corn and coconut oil) from small-scale and family farmings organized in local cooperatives	Over 40% per year of the raw materials used in the production of biodiesel are acquired from family farming strengthening and empowering rural communities and cooperatives	Brazil	Borger and Costa [7]
Strengthening Local Governance for Disaster-Resilient Communities (SAKSHAM)	This project’s goal is to promote community resilience through an integrated approach to disaster risk reduction and management planning, capacity strengthening, and resilient livelihoods	Around 7,000 marginalized farmers will be trained in climate-smart agricultural practices and more than 280 demo plots will be established to demonstrate to neighbouring farmers the benefits of adopting climate-smart agricultural practices	Nepal	LWR [38]
Uptown Pittsburgh EcoInnovation District	Aimed at reviving the community in Uptown Pittsburgh (USA) and bringing members of the community together for environmental projects, this project started in 2015 and emphasizes innovation and deployment of district-scale best practices to create the neighbourhoods of the future—resilient, vibrant, resource-efficient and just	This initiative provided green spaces for the community and facilitated small businesses, evading the problem of gentrification by multiple community-based programmes, such as the Sustainable Small Business Designation, the Sustainable Pittsburgh Restaurant Program, the MLK Community Garden, and Tree Tenders	Uptown Pittsburgh (USA) community	Ghosh et al. [21]
What’s for Dinner?	Engaging freshmen students in experiential learning activities to better understand the socio-economic dynamics of their foodshed in Winona, Minnesota-USA	This initiative reduced barriers to food access while building community through ethnic gardens, food pantries, and knowledge about food production, consumption, and waste	USA	Borsari and Kunnas [8]

shows the case studies in Latin America and the Caribbean, East Africa, North America, Europe, and Asia which were considered in this analysis.

**Table 2:**
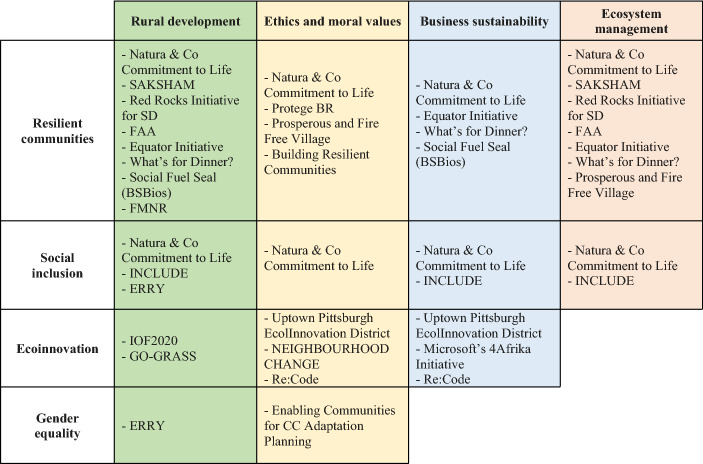
Sustainability topics addressed in the presented case studies

### Framework

The analysis of the case studies provided in Table 2 demonstrates how any given sustainable development project can address multiple social values while addressing relevant sustainability issues.

Figure 2

**Fig. 2:**
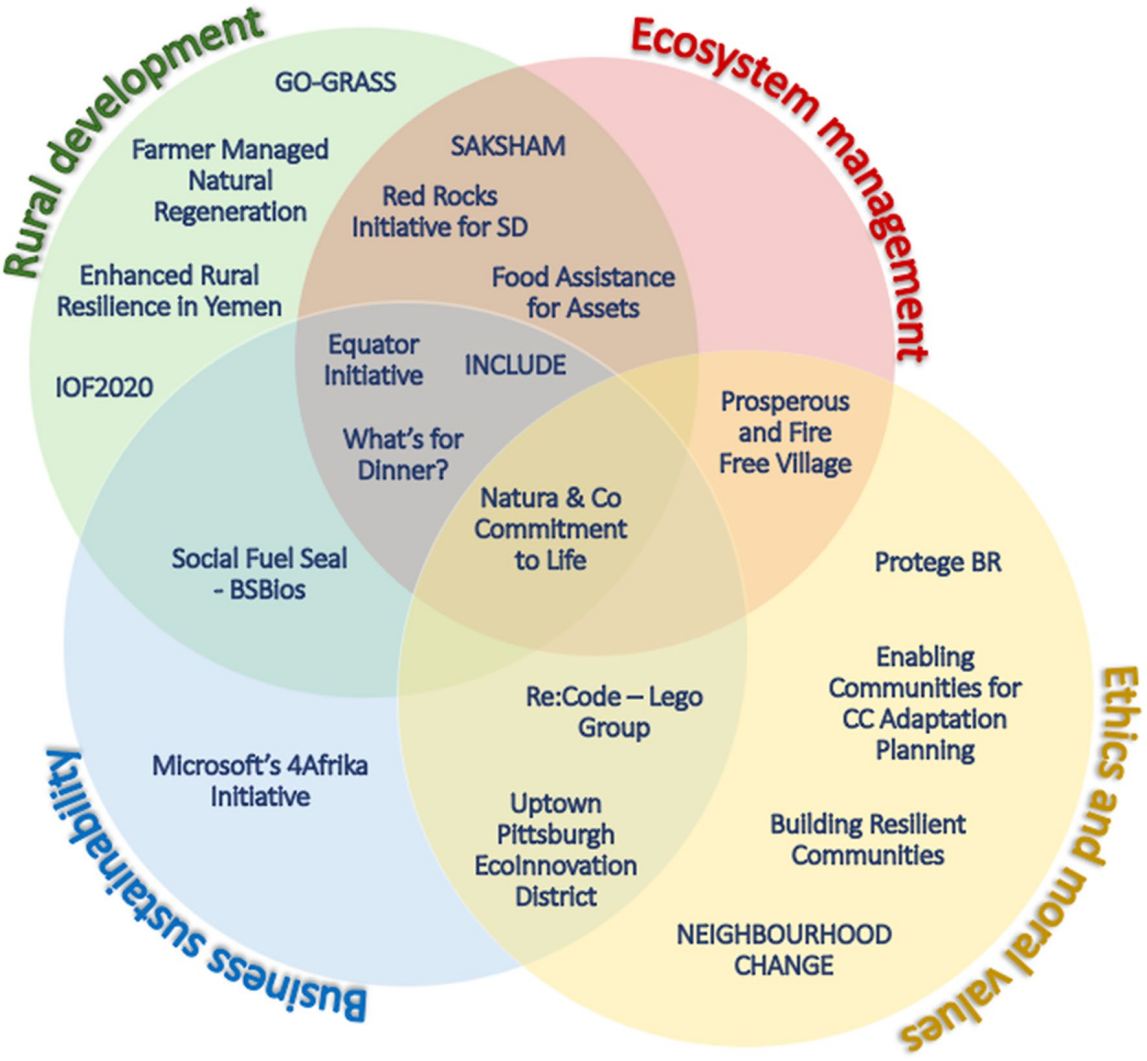
Classification of case studies according to the proposed clusters of social values research topics (red: ecosystem management; green: rural development; blue: business sustainability; yellow: ethics and moral values)

further demonstrates the ways in which the community-based sustainable development projects often intersect multiple social value categories. The presented case studies were distributed across the main clusters identified from the bibliometric analysis. The red one represents the ecosystem management, which aims to protect environmental conditions, the green cluster centres on social values in rural development, the blue group gathers social values regarding business sustainability and Corporate Social Responsibility along with the offshoot of globalization, and the yellow cluster is focused on ethics and moral values (educational, religious and neighbourhood settings).

Most of the case studies addressed social values related to more than one cluster. For instance, the corporate case called Natura and Co Commitment to Life covers strategies focused on business sustainability, rural development, ecosystem management and ethics and moral values. Withal Natura and Co, cases such as SAKSHAM, Red Rocks Initiative, FAA, INCLUDE, Equator Initiative and What’s for Dinner? point out a strong link between social values across rural development and ecosystem protection. Another meaningful string was found among business sustainability, CSR, and ethical values in view of the case studies Re-Code and the EcoInnovation District in Uptown Pittsburgh.

The implications of the results for the implementation of the SDGs are twofold. Firstly, it illustrates that much can be gained by providing an emphasis on social sustainability when it comes to realizing the SDGs. Secondly, whereas the targets of each SDG are quite specific, social sustainability permeates all of them. This includes not only socially oriented SDGs such as SDG1, SDG2, SDG4, or SDG5 for instance, but also some “technical ones” such as SDG11, SDG12, or SDG13, all of which have strong social roots.

### Discussion

This study has demonstrated that social values are being addressed in community-based sustainable development projects, however the social values considered herein differ depending on the focus of each project. The social values may be related, for example, to rural traditions and cultures, or to business stakeholders. Because sustainable development is locally based and context-specific, such that action and solutions are grounded in local needs [58], it follows that the social values considered would be tailored to the presenting issue.

However, it also became clear that currently, there is no a common terminology, nor a description of social values in the context of sustainable development. While the number of papers (74) identified in our bibliometric analysis does provide an insight into the broad arenas in which researchers are exploring sustainable development and social values, we are limited in our ability to draw strong conclusions about the realm of social values research in sustainable development. We note, for example, the lack of frequently used terms related to equity and justice, although we are aware that there are researchers that are exploring these topics in a sustainable development framework. This suggests that some scholars who do this type of research are using alternative terms than those we used in our search string (“sustainable development” and “social values”). For example, they might have used the term “sustainability” instead of “sustainable development” or, instead of using “social values” they used a specific social value concept they focused on, such as equity. Thus, we recommend that future research identifies the way in which specific social values are brought into sustainability and sustainable development narratives, such that future analyses can investigate more thoroughly, the ways in which social values are defined and advanced in sustainable development work.

Furthermore, the case studies that included social values in sustainable development were not taken to a broader analysis level to substantiate whether social values are being engendered to promote sustainability. Are the social values considered in rural development, business sustainability, ecosystem management, and morals and ethics likely to promote a societal basis supportive of change and transformation? Are community-based projects assessing the degree to which there is a change in social values that prioritize consumerism, for example, over social well-being? We posit that while it is essential that social values continue to be assessed and incorporated into community-based sustainable development projects as reported, a more comprehensive effort must be started to analyse the ways in which broader social values are impacting our ability to achieve sustainable development in different places around the world.

## Conclusions

This paper explores the notions of social values in sustainable development, within the context of community experiences. The literature was reviewed and trends related to social values and sustainable development were investigated, through a bibliometric study and juxtaposed to a set of case studies from a variety of community-based projects, with the goal of illustrating the advantages of a focus on social values can bring about in promoting sustainable development. A framework has been presented that links up the bibliometric clusters and the case studies. The evidence gathered valuable data from these analyses and allow some conclusions to be made.

Firstly, the bibliometric study reveals four clusters where the featured values feature relate to sustainable development. This relationship appears in the contexts of:

- Ecosystem management—where social values are evident;
- Rural development—where social values in a rural development setting are apparent;
- Business sustainability—where values are driven by corporate social responsibility and stakeholder values;
- Ethics and moral values about environmental attitudes, human capital, sustainable practices, and a sustainable future.

Secondly, the case studies illustrate that social values are being considered in a variety of projects; the framework deployed to analyse the case studies under the headings identified above, suggests that while community-based sustainable development projects may differ in the values considered, some projects address more than one cluster.

Thirdly, it is apparent that a lack of common terminology in relation to social values in the context of sustainable development is an obstacle to the analysis of the relationship between the two.

Finally, case studies where social values and sustainable development are linked, rarely consider whether social values are being engendered, influenced, or changed as a result of community-based projects. Thus, with less understanding of value change, we may fall short of achieving sustainable development.

The paper has two main implications. The first is that it sheds light on a topic of central relevance, since social components are key elements of sustainability, both as a theme and as an area of knowledge. The second is that the information here compiled and the findings deriving from them provide a timely overview of some of the variables which characterize the extent to which social aspects influence the sustainability debate.

Our research does have some limitations. For instance, it focused on case studies as data collection instruments, and not on other empirical tools such as surveys or interviews. In addition, the range of the case studies is limited to some of the topics identified by the authors, namely rural development, ethics and moral values, business sustainability and ecosystem management. But despite these constraints, the study represents a welcome addition to the literature, in the sense that it has gathered evidence demonstrating how social values under a sustainable development perspective are perceived, and the added value this brings to community experiences.

Moving forward, some measures which may be deployed to better take social values into account, when planning policies or making decisions on spending, which may affect specific groups or communities should be:

- A more systematic approach to taking social values into account when undertaking projects on the principles of sustainable development.
- A greater use of indicators such as public participation and community acceptance, since some social values are not truly objective and—as such—not easy to quantify.
- A more adaptable design and use of a common framework, which may cater for a more accurate measurement of considerations of social values in sustainability projects.
- Employment of innovative models to promote social sustainability issues, both in communities, in schools and workplaces, in particular.

A further measure that could implemented is to design tools, which may cater to an assessment of the impacts of a project, as far as influencing social values are concerned. A due emphasis on social values may allow communities and their stakeholders to understand the advantages of pursuing sustainable development, in a way that they can relate to.

## Data Availability

Not applicable.
